# Prevalence of post-traumatic stress disorder and validity of the Impact of Events Scale – Revised in primary care in Zimbabwe, a non-war-affected African country

**DOI:** 10.1192/bjo.2022.621

**Published:** 2023-02-16

**Authors:** Melanie A. Abas, Monika Müller, Lorna J. Gibson, Sarah Derveeuw, Nirosha Dissanayake, Patrick Smith, Ruth Verhey, Andrea Danese, Dixon Chibanda

**Affiliations:** Centre for Global Mental Health, Institute of Psychiatry, Psychology and Neuroscience, King's College London, UK; Centre for Global Mental Health, Institute of Psychiatry, Psychology and Neuroscience, King's College London, UK; and Clinic for Adult Psychiatry and Psychotherapy, University Hospital of Psychiatry Bern, Switzerland; Centre for Global Mental Health, London School of Hygiene and Tropical Medicine, UK; Department of Child and Adolescent Psychiatry, Institute of Psychiatry, Psychology & Neuroscience, King's College London, UK; Friendship Bench, Harare, Zimbabwe; Department of Child and Adolescent Psychiatry, Institute of Psychiatry, Psychology & Neuroscience, King's College London, UK; Social, Genetic and Developmental Psychiatry Centre, Institute of Psychiatry, Psychology & Neuroscience, King's College London, UK; and National and Specialist CAMHS Clinic for Trauma, Anxiety, and Depression, South London and Maudsley NHS Foundation Trust, UK; Centre for Global Mental Health, London School of Hygiene and Tropical Medicine, UK; Friendship Bench, Harare, Zimbabwe; and Department of Psychiatry, University of Zimbabwe, Zimbabwe

**Keywords:** Global mental health, psychological testing, low- and middle-income countries, post-traumatic stress disorder, rating scales

## Abstract

**Background:**

A critical step in research on the epidemiology of post-traumatic stress disorder (PTSD) in low-resource settings is the validation of brief self-reported psychometric tools available in the public domain, such as the Impact Event Scale – Revised (IES-R).

**Aims:**

We aimed to investigate the validity of the IES-R in a primary healthcare setting in Harare, Zimbabwe.

**Method:**

We analysed data from a survey of 264 consecutively sampled adults (mean age 38 years; 78% female). We estimated the area under the receiver operating characteristic curve and sensitivity, specificity and likelihood ratios for different cut-off points of the IES-R, against a diagnosis of PTSD made using the Structured Clinical Interview for DSM-IV. We performed factor analysis to evaluate construct validity of the IES-R.

**Results:**

The prevalence of PTSD was 23.9% (95% CI 18.9–29.5). The area under the curve for the IES-R was 0.90. At a cut-off of ≥47, the sensitivity of the IES-R to detect PTSD was 84.1 (95% CI 72.7–92.1) and specificity was 81.1 (95% CI 75.0–86.3). Positive and negative likelihood ratios were 4.45 and 0.20, respectively. Factor analysis revealed a two-factor solution, with both factors showing good internal consistency (Cronbach's factor-1 *α* = 0.95, factor-2 *α* = 0.76). In a *post hoc* analysis, we found the brief six-item IES-6 also performed well, with an area under the curve of 0.87 and optimal cut-off of 15.

**Conclusions:**

The IES-R and IES-6 had good psychometric properties and performed well for indicating possible PTSD, but at higher cut-off points than those recommended in the Global North.

Recognition and treatment of depression and anxiety has been a major focus of the global mental health movement.^1^ Post-traumatic stress disorder (PTSD) has received relatively little attention in primary healthcare in low-resource settings, except in regions affected by war or natural disaster.^2,3^ A systematic review in primary healthcare settings reported a median point prevalence of diagnostic interview-ascertained PTSD of 12.5% (range: 2–32.5%), with significant heterogeneity.^4^ Only two of the 41 studies included were from a low-resource setting, both of which were from South Africa.^5,6^ PTSD might be more common in primary healthcare in low-resource compared with high-resource settings, given socioeconomic inequalities and exposure to intimate partner violence,^7^ infant mortality and death of loved ones following accidents.^8^

A critical step for research and service development for PTSD in low-resource settings is the validation of brief self-reported tools that are available in the public domain. The Impact of Events Scale – Revised (IES-R) is a freely available self-report questionnaire to assess subjective intrusion, avoidance and hyperarousal indicative of possible PTSD after a traumatic event.^9^ It does not strictly follow the diagnostic criteria of the DSM-IV or DSM-5, and the validity of its three factor structure has been questioned in favour of the original two-factor model.^10,11^ In Sub-Saharan Africa, its use has been reported in The Gambia, Ghana, Rwanda, Democratic Republic of Congo and Uganda.^2,12–16^ All but one of these studies took place in communities affected by armed violence, war or genocide, leaving a gap in validation studies in non-conflict settings in the southern African region. Moreover, none of these studies measured the tool's criterion validity using sensitivity, specificity, likelihood ratios and receiver operating characteristic (ROC) curves. The primary aim of the present study was to describe the criterion and construct validity of the IES-R and the brief six-item version (IES-6) in people using general primary healthcare in Zimbabwe, as compared with a diagnosis of PTSD based on the Structured Clinical Interview for DSM-IV (SCID-IV).

## Method

### Study design and setting

Zimbabwe is a lower-middle-income country with a population of 15 million. Between 34 and 49% of the population live in extreme poverty, defined as living on <$1.90 per day.^17^

Zimbabwe has one of the highest HIV prevalences in Sub-Saharan Africa, at 12.9%.^18^ We analysed pre-existing data from a cross-sectional survey that had taken place at a large primary healthcare centre in Mbare. The original survey had been conducted to validate the Patient Health Questionnaire-9 for depression and the Shona Symptom Questionnaire for common mental disorders.^19^

Mbare is the oldest high-density suburb in the southern district of the capital city of Harare. The predominant language of its population is Shona, followed by Ndebele and English. Mbare is characterised by high levels of deprivation, unemployment, mobility and crime.^20^ Many households lack adequate electric, water and sanitation services. The major determinants of poverty in high-density suburbs in Harare are large family size, low education level of the household head, lack of income from permanent employment, low cash transfers and short length of residence in the suburb.^21^ The primary healthcare centre caters to a catchment population of 200 000. It has an average attendance of 140 patients per day and provides a range of services, including acute primary care, chronic disease out-patients, family planning, maternity, and services for the prevention and treatment of HIV and tuberculosis.

### Participants

Adults aged ≥18 years attending the Mbare primary healthcare centre, and seeking healthcare for themselves for any reason, were invited to take part during a 2-week period in 2013. Women in the last trimester of pregnancy or first 6 weeks post-natal and those deemed by clinic triage staff to be in need of urgent clinical care were excluded. The authors assert that all procedures contributing to this work comply with the ethical standards of the relevant national and institutional committees on human experimentation and with the Helsinki Declaration of 1975, as revised in 2008. All procedures involving human patients were approved by the Medical Research Council of Zimbabwe (approval number MRCZ/A/1732) and the London School of Hygiene and Tropical Medicine (approval reference 8457). Written informed consent was obtained from all participants, and they were compensated with US$3 for their participation, which covered transport costs.

### Procedure

People presenting at Mbare primary healthcare centre were entered onto a register by the reception clerk. This register was used to develop the sampling frame for the study. Each day, sixty patients were randomly selected from the register, based on a computer-generated random number. Those eligible and willing to take part were interviewed by one of four trained research assistants.

Each research assistant was responsible for collecting written informed consent, interviewing participants to collect sociodemographic characteristics and health information and administering the IES-R. Paper and pencil was used for data collection. Data were double entered onto a password-protected database. Participants were then interviewed separately by one of four trainee psychiatrists to diagnose PTSD, using the SCID-IV. The psychiatrists were blinded to the IES-R screening results. Data collection took place in a quiet space specifically designated for the study team, to avoid inference with the normal primary healthcare clinical activities and maintain privacy. The administration of the IES-R and collection of sociodemographic and health-related data by the research assistant lasted for 20–30 min, and the evaluation of the PTSD diagnosis by a psychiatrist using the SCID-IV took around 45 min. The trainee psychiatrists had between 1–2 years postgraduate experience in psychiatry and had attended a 2-week training on the SCID-IV, including its use in the Shona language, led by a senior local psychiatrist (D.C.). The training included providing information about diagnostic criteria of PTSD and role-plays during a pilot assessment of four patients, using the SCID-IV interview.

The team who translated the IES-R from English to Shona comprised a bilingual consultant

psychiatrist (D.C.), a bilingual psychologist and five lay health workers each with over 6 years of experience providing mental health counselling.^19^ The psychiatrist carried out the first forward translation from English into the local language of Shona. To ensure equivalence to the original version as well as accounting for contextual use of local terms for mental distress, this first Shona translation was reviewed by the rest of the translation team. The resulting second Shona version was back-translated into English by an independent language expert from the University of Zimbabwe, with final approval of the back-translated version by the translation team. Discrepancies were resolved by consensus. None of the IES-R items were modified to an extent that equivalence to the original version was lost. The SCID-IV was already available and in use in Zimbabwe, having been previously translated using the same approach to what we describe here for the IES-R translation. The questionnaire to collect sociodemographic variables was originally designed in Shona.

### Measures

#### Sociodemographic information

Age, gender, education, marital status, employment status, household income and HIV status were collected through a questionnaire developed for similar studies previously conducted in this setting.^22^

#### The IES-R and IES-6

The IES-R is a 22-item self-report measure that is used to screen for PTSD and monitor outcomes through assessment of subjective distress.^9^

Respondents are told they will be shown a list of difficulties people sometimes have after stressful life events. They are asked to score how much they were distressed or bothered in the past 7 days, with respect to a specific stressful event that they are asked to specify; for example, ‘Any reminders brought back feelings about it?’. Each difficulty is self-rated on a five-point scale ranging from 0 (‘not at all’) to 4 (‘extremely’) distressed or bothered. The score ranges from 0 (no symptoms of PTSD) to 88 (severe and frequent symptoms of PTSD). The 22 symptoms are grouped into three subscales, which reflect the three core sets of symptoms of PTSD, i.e. intrusive memories (eight items), avoidance (eight items) and hyperarousal (six items). High levels of internal consistency have been previously reported (intrusion: Cronbach's *α* = 0.87–0.94; avoidance: Cronbach's *α* = 0.84–0.87; hyperarousal: Cronbach's *α* = 0.79–0.91) and factor loadings across the three domains range from 0.52 to 0.92.^9,10^ In the USA and UK, the recommended cut-off point is 33.^9,10^

The IES-6 comprises six out of the 22 items of the IES-R from the three subscales.^23^ It has shown good correlation with IES-R subscales and has a high area under the ROC curve (AUC), comparable to the AUC for the IES-R, using the PTSD Checklist as the gold standard.^23^

#### Assessment of traumatic events

We used a simple binary scale that inquiries about the experience of traumatic events, including rape, assault, road traffic accidents, experiences of witnessing violent acts perpetrated against close others and deaths of close family members. The last author (D.C.), a Zimbabwean psychiatrist, cross-culturally adapted this tool from the Life Events and Difficulties Schedule.^22,24^

#### Diagnosis of PTSD using the SCID-IV

Diagnosis of PTSD was made according to the diagnostic criteria published in the DSM-IV,^25^ using the SCID-IV.^26^

This semi-structured interview consists of probe questions and follow-up questions to guide the assessment, allowing clinicians to make their diagnosis during the course of the interview without a formal scoring algorithm or programme.

### Data analysis

We calculated the prevalence of PTSD, based on the SCID-IV, with corresponding 95% confidence intervals, using a binominal distribution that allows for modelling of binary outcomes. There were no missing data in the diagnostic variables (SCID, IES-R).

Age was missing for ten of the participants (3.8%) and HIV status was not known for 27 participants (10.2%). All other sociodemographic variables were complete. We compared sociodemographic characteristics of patients with and without PTSD by using *χ*^2-^test, and excluded patients with missing data.

To assess the criterion validity of the Shona version of the IES-R, we estimated the AUC for the IES-R against the gold-standard clinical diagnosis based on the SCID. We prespecified an AUC of >0.80 as clinically relevant. We calculated sensitivities, specificities, positive and negative predictive values and likelihood ratios for different cut-off points, to identify potential cut-off points to indicate likely PTSD. The positive and negative likelihood ratios directly relate sensitivity and specificity [positive likelihood ratio = sensitivity/(1–specificity); negative likelihood ratio = (1–sensitivity)/specificity], making them useful parameters to evaluate the clinical usefulness of a diagnostic test. A cut-off point is considered to provide clinically relevant power to rule in or out a diagnosis if the positive LR is >5 and negative LR is <0.2.^27^ We also calculated the Youden Index, another method to identify an appropriate cut-off point.^28^ We performed a *post hoc* sensitivity analysis of the criterion validity for the IES-6 following the same statistical procedures as for the IES-R.

To assess the construct validity of the Shona version of the IES-R, we conducted an exploratory principal axis factor analysis with oblimin rotation on the entire sample. We used Bartlett's test of sphericity to ensure items within the scale were significantly correlated. The test showed a significant result (*χ*^2^(231) = 3436.02, *P* < 0.001).^29^ The Kaiser-Meyer-Olkin (KMO) measure of sampling adequacy was applied to ensure items shared sufficient variance to justify factor analysis. Sampling adequacy was determined to be high (KMO = 0.948) as suggested by Hair and colleagues, who consider sampling adequacy values of ≥0.80 to be excellent.^30^ We applied the Kaiser–Guttman criterion for factor retention (i.e. retained factors with Eigenvalues >1. Salience was detected by applying item retention criteria as described by Tabachnick and Fidell:^31^ (a) factor loadings of at least 0.40 on any factor retained, ensuring a high degree of association between the item and the factor; (b) a difference of at least 0.30 between the loading on the primary factor and the loading on other factors and (c) a minimum of three items per factor. Internal consistencies within the scale were calculated with Cronbach's *α* coefficient. Factor score correlations were assessed with Pearson's correlation coefficient. All analyses were conducted with Stata for Windows version 14.2 (StataCorp, College Station, Texas, USA).

## Results

### Study population

Of the 332 people approached during the study period, 297 were eligible. Of those eligible, 264 (88.9%) gave consent to take part. The sample was predominantly female (*n* = 208; 78.8%) and cohabiting (*n* = 157; 59.5%), with a mean age of 37.6 years (s.d. 9.69). Three quarters (*n* = 199; 75.4%) had at least entered secondary education, 59 (22.3%) had entered primary education only and six (2.3%) had no education. Nearly half were unemployed (*n* = 112; 42.4%) or casually or self-employed (*n* = 129; 48.9%), with only a minority employed (*n* = 26; 9.8%). A monthly household income of US$200 or less was reported by 81.1% (*n* = 214). Regarding HIV status, 254 stated that they knew their HIV status, of whom 165 (70.0%) self-reported to be HIV-positive.

### PTSD prevalence

The overall prevalence of PTSD diagnosed based on the DSM-IV was 23.9% (95% CI 18.9–29.5). The most frequent stressful events described by those with PTSD symptoms were death of a close family member (partner, child or other close relative) (58.7%), being assaulted (physically or sexually) (52.4%) and other types of violence, including witnessing violence to a loved one (15.9%). Most participants with PTSD (63.5%) reported psychological symptoms after one traumatic event, but 23.8% reported experiencing two traumatic events and 12.7% reported experiencing three or more traumatic events. As shown in Table 1, those with PTSD were more likely to be living with HIV than not living with HIV, and be widowed or divorced rather than married or having a partner, compared with those without PTSD. For those who knew they were living with HIV (*n* = 165), the prevalence of PTSD was 32.1% (95% CI 25.1–39.8), and for those who knew their HIV status was negative (*n* = 72), the prevalence of PTSD was 12.5% (95% CI 5.9–22.4).

**Table 1:** Demographic and health characteristics by post-traumatic stress disorder status

	PTSD positive (*n* = 63)		PTSD negative (*n* = 201)		
	*n*	%	*n*	%	*P*-value *χ*^2-^test
Gender					0.898
Male	13	20.6%	43	21.4%	
Female	50	79.4%	158	78.6%	
Age group, years					0.292
<30	11	17.5%	47	24.6%	
30–39	21	33.3%	70	36.7%	
≥40	31	49.2%	74	38.7%	
Marital status					0.048
Married/partner	31	49.2%	126	62.7%	
Divorced/widowed	19	30.2%	33	16.4%	
single	13	20.6%	42	20.9%	
Education					0.131
Primary or less	11	17.5%	54	26.9%	
Secondary or more	52	82.5%	147	73.1%	
Current employment status					0.527
Unemployed	29	46.0%	83	41.3%	
Permanent (full/part time)	4	6.4%	22	11.0%	
Casual/self-employed	30	47.6%	96	47.8%	
Household monthly income					0.221
≤$50	30	47.6%	68	33.8%	
$51–100	10	15.9%	37	18.4%	
$101–200	12	19.1%	57	28.4%	
>$200	11	17.5%	39	19.4%	
HIV status					0.002
Negative	9	14.5%	63	36.0%	
Positive	53	85.5%	112	64.0%	

PTSD, post-traumatic stress disorder.

### Performance of the IES-R and IES-6 against a diagnosis of PTSD using the SCID-IV

**Fig. 1:**
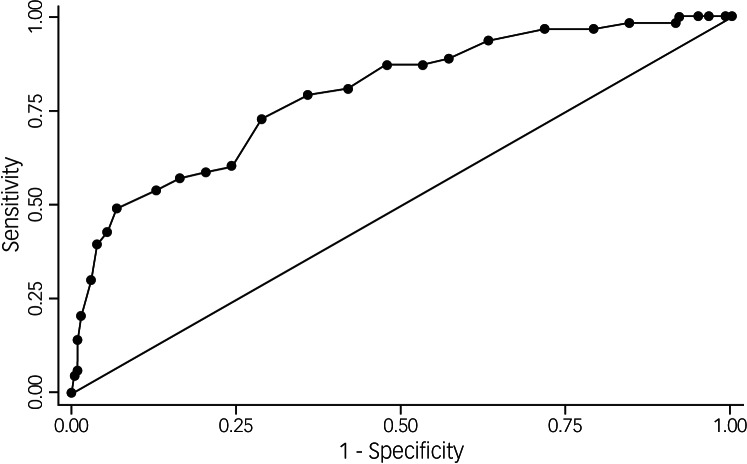
Receiver operating characteristic curve for Impact of Events Scale – Revised as compared with the clinical diagnosis of post-traumatic stress disorder according to the Structured Clinical Interview for DSM-IV.

Figure 1 shows the ROC curve for the IES-R, with an AUC of 0.90. Table 2 presents measures of criterion validity at different cut-off points of the IES-R. At the standard cut-off point of ≥ 3, the IES-R had a sensitivity of 98.4% (95% CI 91.5–100.0) and a low specificity of 51.2% (95% CI 44.1–58.3).

**Table 2:** Criterion validity of the Impact of Events Scale – Revised according to different cut-off point values as compared with the clinical diagnosis of post-traumatic stress disorder according to the Structured Clinical Interview for DSM-IV

Cut-off value IES-R^a^	Number of patients				Sensitivity, % (95% CI)	Specificity, % (95% CI)	Likelihood ratio	
	True positive	False negative	False positive	True negative			Positive	Negative
31	63	0	110	91	100.0 (94.3–100.0)	45.3 (38.3–52.4)	1.83	0.00
32	62	1	104	97	98.4 (91.5–100.0)	48.3 (41.2–55.4)	1.90	0.03
33	62	1	98	103	98.4 (91.5–100.0)	51.2 (44.1–58.3)	2.02	0.03
34	62	1	93	108	98.4 (91.5–100.0)	53.7 (46.6–60.8)	2.13	0.03
35	62	1	88	113	98.4 (91.5–100.0)	56.2 (49.1–63.2)	2.25	0.03
36	61	2	83	118	96.8 (89.0–99.6)	58.7 (51.6–65.6)	2.34	0.05
37	61	2	73	128	96.8 (89.0–99.6)	63.7 (56.6–70.3)	2.67	0.05
38	61	2	70	131	96.8 (89.0–99.6)	65.2 (58.2–71.7)	2.78	0.05
39	61	2	69	132	96.8 (89.0–99.6)	65.7 (58.7–72.2)	2.82	0.05
40	59	4	66	135	93.7 (84.5–98.2)	67.2 (60.2–73.6)	2.85	0.09
41	59	4	63	138	93.7 (84.5–98.2)	68.7 (61.8–75.0)	2.99	0.09
42	58	5	60	141	92.1 (82.4–97.4)	72.3 (65.5–78.5)	3.32	0.11
43	58	5	54	147	92.1 (82.4–97.4)	73.1 (66.4–79.1)	3.43	0.11
44	57	6	52	149	90.5 (80.4–96.4)	74.1 (67.5–80.0)	3.50	0.13
45	57	6	47	154	90.5 (80.4–96.4)	76.6 (70.1–82.3)	3.87	0.12
46	56	7	44	157	88.9 (78.4–95.3)	78.1 (71.7–83.6)	4.06	0.14
47	53	10	38	163	84.1 (72.7–92.1)	81.1 (75.0–86.3)	4.45	0.20
48	51	12	35	166	81.0 (69.1–89.8)	82.6 (76.6–87.6)	4.65	0.23
49	51	12	34	167	81.0 (69.1–89.8)	83.1 (77.2–88.0)	4.79	0.23
50	51	12	31	170	81.0 (69.1–89.8)	84.6 (78.8–89.3)	5.25	0.23

Values are point estimates with corresponding 95% confidence intervals. Positive likelihood ratio >5 and negative likelihood ratio <0.2 indicate clinically relevant cut-off point. IES-R, Impact of Events Scale – Revised; PTSD, post-traumatic stress disorder.

a. IES-R: 0 = no symptoms of PTSD, 88 = severe and frequent symptoms of PTSD. Cut-off: below cut-off was considered as test positive; equal or above cut-off was considered as test negative.

The positive and negative likelihood ratios were 2.0 and 0.03, respectively. At a cut-off point of 47, the sensitivity and specificity were 84% and 81%, respectively, with a balance of strong positive and negative likelihood ratios. The Youden Index suggested a cut-off point of 45; this cut-off point yielded a sensitivity of 90.5% (95% CI 80.4–96.4), but when compared with the cut-off point of 47, we found a lower specificity of 76.6% (95% CI 70.1–82.3) and less optimal positive and negative likelihood ratios of 3.87 and 0.12, respectively.

**Fig. 2:**
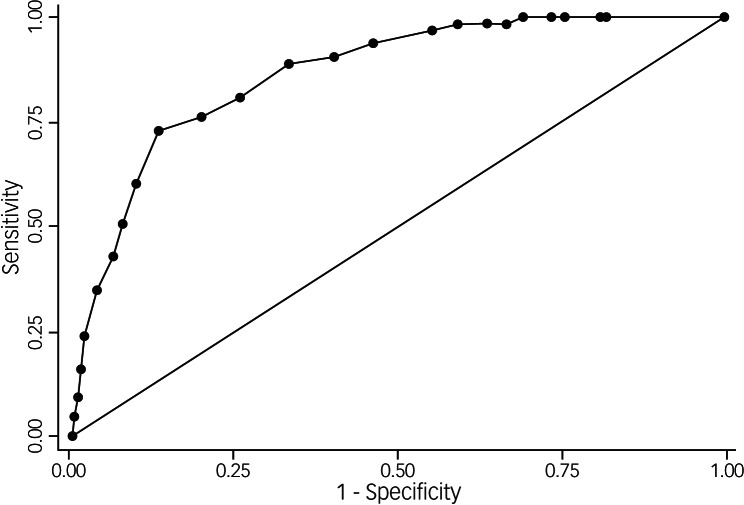
Receiver operating characteristic curve for the brief six-item Impact of Events Scale as compared with the clinical diagnosis of post-traumatic stress disorder according to the Structured Clinical Interview for DSM-IV.

Figure 2 shows the ROC curve for the IES-6, with an AUC of 0.87. Table 3 shows sensitivity, specificity and positive and negative likelihood ratios for different cut-off points of the IES-6. The Youden Index suggested a cut-off point of 15 as the ideal cut-off point for the IES-6 to screen for PTSD, which is in keeping with the balance of likelihood ratios at this cut-off point, as shown in Table 3.

**Table 3:** Criterion validity of the six-item Impact of Events Scale according to different cut-off point values, as compared with the clinical diagnosis of post-traumatic stress disorder according to the Structured Clinical Interview for DSM-IV

Cut-off value IES-6^a^	Number of patients				Sensitivity, % (95% CI)	Specificity, % (95% CI)	Likelihood ratio	
	True positive	False negative	False positive	True negative			Positive	Negative
5	63	0	139	62	100.0 (94.3–100.0)	30.8 (24.5–37.7)	1.45	0.00
6	62	1	134	67	98.4 (91.5–100.0)	33.3 (26.9–40.3)	1.48	0.05
7	62	1	128	73	98.4 (91.5–100.0)	36.3 (29.7–43.4)	1.55	0.04
8	62	1	119	82	98.4 (91.5–100.0)	40.8 (33.9–47.9)	1.66	0.04
9	61	2	111	90	96.8 (89.0–99.6)	44.8 (37.8–51.9)	1.75	0.07
10	59	4	93	108	93.7 (84.5–98.2)	53.7 (46.6–60.8)	2.02	0.12
11	57	6	81	120	90.5 (80.4–96.4)	59.7 (52.6–66.5)	2.25	0.16
12	56	7	67	134	88.9 (78.4–95.4)	66.7 (59.7–73.1)	2.67	0.17
13	51	12	52	149	81.0 (69.1–4.07)	74.1 (67.5–80.0)	3.13	0.26
14	48	15	40	161	76.2 (63.8–86.0)	80.1 (73.9–85.4)	3.83	0.30
15	46	17	27	174	73.0 (60.3–83.4)	86.6 (81.1–91.0)	5.44	0.31
16	38	25	20	181	60.3 (47.2–72.4)	90.0 (85.1–93.8)	6.06	0.44
17	32	31	16	185	50.8 (37.9–63.6)	92.0 (87.4–95.4)	6.38	0.53
18	27	36	13	188	42.9 (30.5–56.0)	93.5 (89.2–96.5)	6.63	0.61
19	22	41	8	193	34.9 (23.3–48.0)	96.0 (92.3–98.3)	8.77	0.68
20	15	48	4	197	23.8 (14.0–36.2)	98.0 (95.0–99.5)	11.96	0.78

Values are point estimates with corresponding 95% confidence intervals. Positive likelihood ratio >5 and negative likelihood ratio <0.2 indicate clinically relevant cut-off point. IES-6, brief six-item Impact of Events Scale; PTSD, post-traumatic stress disorder.

a. IES-6: 0 = no symptoms of PTSD, 24 = severe and frequent symptoms of PTSD. Cut-off: below cut-off was considered as test positive; equal or above cut-off was considered as test negative.

### Exploratory factor analysis for the IES-R

Exploratory factor analysis revealed that the Shona version of the IES-R had two underlying factors accounting for 74.52% of the total variance. Factor 1 showed an Eigenvalue of 10.29 and factor 2 showed an Eigenvalue of 6.10. A combination of intrusion and hyperarousal items (items 1, 2, 3, 4, 6, 7, 9, 10, 12, 14, 15, 16, 18, 19, 20 and 21) loaded onto factor 1, and six of the eight avoidance items (items 5, 8, 11, 13, 17, and 22) loaded onto factor 2. Factor loadings per scale item are detailed in Table 4, with loadings onto factor 1 ranging from 0.58 to 0.86 and loadings onto factor 2 ranging from 0.52 to 0.81. We calculated a Cronbach's *α* of 0.95 for the whole scale, *α* = 0.95 for factor 1 and *α* = 0.76 for factor 2. Intercorrelations between subscale scores were *r* = 0.43 (*P* < 0.001).

**Table 4:** Factor loadings for 22 items of the Shona version of the Impact of Events Scale – Revised (IES-R), according to the exploratory factor analysis

Item		Factor 1	Factor 2
1	Any reminder brought back feelings^a^	0.77	
2	I had trouble staying asleep^a^	0.74	
3	Other things kept making me think about it^a^	0.79	
4	I felt irritable and angry^b^	0.76	
5	I avoided letting myself get upset about it^c^		0.63
6	I thought about it when I didn't mean to^a^	0.71	
7	I felt as if it hadn't happened or wasn't real^c^	0.58	
8	I stayed away from reminders of it^c^		0.50
9	Pictures about it popped into my mind^a^	0.86	
10	I was jumpy and easily startled^b^	0.84	
11	I tried not to think about it^c^		0.81
12	I was aware that I still had a lot of feelings about it, I didn't deal with them^c^	0.71	
13	My feelings about it were kind of numb^c^		0.52
14	I found myself acting or feeling like I was back at that time^a^	0.75	
15	I had trouble falling asleep^b^	0.70	
16	I had waves of strong feelings about it^a^	0.81	
17	I tried to remove it from my memory^c^		0.77
18	I had trouble concentrating^b^	0.63	
19	Reminders of it caused me to have physical reactions, such as sweating, trouble breathing, nausea or a pounding heart^b^	0.79	
20	I had dreams about it^a^	0.69	
21	I felt watchful and on-guard^b^	0.72	
22	I tried not to talk about it^c^		0.61

IES-R, Impact of Events Scale – Revised.

a. Items referring to the core symptom of intrusion, according to the IES-R.

b. Items referring to the core symptom of hyperarousal, according to the IES-R.

c. Items referring to the core symptom of avoidance, according to the IES-R.

## Discussion

This is the first study from any African country to report on both criterion and construct validity of the IES-R, and the first to validate the IES-R in primary healthcare in a non-conflict-affected African country. The IES-R performed well in this setting, similar to that reported in studies of its criterion validity in other countries.^10,32,33^ None of the cut-off points achieved the ideal of a positive likelihood ratio above 5 and a negative likelihood ratio below 0.2,^27^ but they were acceptable at cut-off points between 46 and 51. The Youden Index suggested a cut-off point of 45. These findings indicate that the recommended cut-off point for the IES-R in this setting is higher than that recommended in the Global North.^9^ This may be a result of high baseline stress, socioeconomic inequalities, high prevalence of HIV (which is associated with PTSD) and the poor access to mental healthcare experienced in Sub-Saharan Africa. These factors are likely to translate to higher stress load, increasing the cut-off point needed to detect probable PTSD.^20,21^ However, variation has also been found in validation studies from high-income countries, with IES-R cut-off points ranging from 22 to 44, with a generally accepted standard cut-off of 33.^34–37^ It is hypothesised that the optimal cut-off score may depend on the type of trauma as well as the time elapsed since the trauma.^37^ Further research on PTSD in low-resource settings should focus on the phenomenology of trauma. The phenomenology should be sensitive to sociocultural differences so that a traumatic event would be considered as such by the majority of the population in a specific country. For example, witnessing of death of a loved one from natural non-violent causes, such as a heart attack, is considered stressful in Zimbabwe, although such an event is not considered to be a severe trauma according to diagnostic manuals established in high-income countries. In Sub-Saharan African countries such as Zimbabwe, death is commonly attributed to supernatural causes, which can have fearful implications for family members left behind.^38,39^ Also, given weak health and social welfare systems, deaths can occur with very limited explanation, adding to stress in bereaved relatives, and death can leave bereaved families with extreme financial and legal difficulties.^40^ The brief IES-6 also had good psychometric properties and performed well in discriminating between cases and non-cases of PTSD. This is promising given its potential applicability in a low-resource setting, where health workers are overburdened.

We found a prevalence of PTSD of 23.9%, based on a SCID (DSM-IV) diagnosis, which suggests that PTSD was common in adults seeking primary healthcare in this low-resource, non-conflict-affected African setting. Our prevalence was at the upper end of the range estimated by the most recent systematic review of PTSD in primary care (range 2–32.5%).^4^ The only two studies from low-income countries included in this systematic review were both from South Africa, with a reported prevalence of 29.6%^5^ and 19.9%,^6^ respectively. The high prevalence we found could be explained by the high frequency of traumas and high baseline psycho-social stress in high-density settlements in Sub-Saharan Africa, which are characterised by extreme poverty, food scarcity and overcrowded housing.^20,21^ Lack of strong systems to address gender-based violence and little access to treatment of PTSD in in low-resource settings will add to the high prevalence.^7^ Many of our participants were living with HIV, which is another reason for the high prevalence of PTSD we reported, as 32.1% of those living with HIV in our sample were diagnosed with PTSD. A systematic review found the overall prevalence of PTSD in persons living with HIV to be 28%, nearly seven times the prevalence in the general population.^41^ We need to consider whether the high prevalence we found could be a result of applying DSM-IV rather than DSM-5 criteria. Although there are a number of important differences between DSM-IV and DSM-5 criteria, including the removal of non-immediate life-threatening illness, such as terminal cancer or HIV, as a qualifying trauma in the DSM-5, these have made only small changes to prevalence estimates.^42,43^ In a similar primary care setting in Zimbabwe, Verhey et al found the prevalence of PTSD to be 20% when using DSM-5 criteria,^44^ which is similar to the prevalence of 23.9% we found using the SCID for DSM-IV. Thus, we do not think our use of DSM-IV rather than DSM-5 criteria explains the high prevalence that we found.

We used exploratory rather than confirmatory factor analysis for this first cross-cultural validation of the newly translated Shona version of the IES-R, which is considered common practice when assessing its validity in a completely new language and research setting.^45,46^ We found high internal consistency of the IES-R, good support for a two-factor solution (one of mostly intrusion items and one of avoidance) and good to high internal consistency of the two factors. A functional relationship has been proposed between intrusion and avoidance, whereby avoidance helps the individual to regulate negative affect generated by intrusive trauma reminders.^47,48^ Hyperarousal as a symptom cluster was introduced more recently when the Impact of Events Scale was revised.^49^ Our results suggest that the Shona version of the IES-R shows greater similarity to the earlier intrusion-avoidant model of PTSD, where hyperarousal and intrusion are not discriminant clusters, but rather consist of items that are highly correlated with one another. This is concurrent with results from other culturally validated samples from the UK, USA, Gambia and Ghana.^10,13,16,50,51^

The strengths of this study include the sample of nearly 300 consecutively sampled attenders, enabling good precision around the estimates. A further strength was that the gold standard for establishing criterion validity for PTSD was diagnosed through a face-to-face interview performed by Zimbabwean clinicians trained to use the SCID-IV. This is commonly used for criterion validation in Zimbabwe, with local mental health specialists trained in its use.^19^ All clinicians making a diagnosis had between 1 and 4 years of specialist training in psychiatry. The validation procedure was preceded by translation and back translation of the IES-R by a team that included bilingual local clinicians and an independent language expert from the University of Zimbabwe, which is another strength of the study. Additionally, we followed recommended methods for validation of a screening tool, which includes the evaluation of its sensitivity, specificity, likelihood ratios and ROC curves to determine the ideal cut-off point for a new setting. Previous research on the IES-R on the African continent did not follow this approach. The large-scale use of the IES-R as screening tool in primary care of a resource-limited setting might be compromised by its length. Therefore, we also evaluated the diagnostic performance of the abbreviated IES-6, which consists of only six items.^23^ We consider the co-evaluation of both PTSD assessment tools as major strengths of our paper. One limitation of our study is that we did not use a validated tool to collect data on traumatic events. The main reason for this was the lack of such validated tools for a non-conflict-affected Zimbabwean primary care setting. However, we based the selection of traumatic events likely to be encountered in this setting on research on life events in Zimbabwe. The last author (D.C.), a Zimbabwean psychiatrist, cross-culturally adapted this tool from the Life Events and Difficulties Schedule.^24,52^ This simple binary scale inquiries about the experience of traumatic events, including rape, assault, road traffic accidents and death of a close family member, which are the traumatic events most likely to be associated with PTSD in non-conflict settings.^53^ A further limitation of our research is that we used the SCID for DSM-IV and not for DSM-5, as the latter tool has not been translated in Zimbabwe.

To conclude, our findings strongly support use of the IES-R in Zimbabwe to detect distress following traumatic events. Use of the IES-R and IES-6 may be of interest to those providing care for people at high risk of PTSD in Zimbabwe, such as victims of sexual violence, people living with HIV, those who have experienced sudden unnatural deaths of loved ones and, considering the present situation, those exposed to severe trauma in the context of COVID-19. The robust methods used for this validation study argue for generalisability to other countries in the region, especially those with high HIV burden. The demonstration of the validity of an avoidance factor in this context supports exploration of therapies for PTSD based on exposure.

## Data Availability

The data that support the findings of this study are available from the corresponding author, M.M., upon reasonable request.
